# Comprehensive landscape and oncogenic role of extrachromosomal circular DNA in malignant biliary strictures

**DOI:** 10.1186/s13578-025-01361-6

**Published:** 2025-02-07

**Authors:** Zhuo Cheng, Xuanmei Luo, Wenzheng Liu, Xiaofang Lu, Hong Chang, Yingchun Wang, Wei Zheng, Xiue Yan, Yonghui Huang

**Affiliations:** 1 Department of Gastroenterology and Hepatology, Peking University Third Hospital, Beijing, 100191 China; 2 Peking University Fifth School of Clinical Medicine, Beijing, China; 3 Department of Gastroenterology, Beijing Tsinghua Changgung Hospital, Beijing, 102218 China

**Keywords:** Malignant biliary strictures, Bile cell-free extrachromosomal circular DNA, Diagnostic biomarkers, Liquid biopsy assay, Nanopore sequencing, Carcinogenesis

## Abstract

**Background:**

Extrachromosomal circular DNA (eccDNA) is crucial for carcinogenesis and bile has direct contact with malignant biliary strictures, yet eccDNA features in bile and its function in malignant biliary strictures remain underexplored.

**Results:**

We observed the widespread presence of eccDNA in bile and systematically profiled the landscape of bile cell-free eccDNA (bcf-eccDNA). For functional exploration, a simple and efficient workflow was designed to synthesize large eccDNA particularly containing multiple regions. Compared with the noncancer group, bcf-eccDNAs in the cancer group had different origins and larger sizes with six characteristic peaks. These peaks were also identified in the validation cohort (100%). There were more bcf-eccDNA carrying *LINC00598* or *CELF2* in malignant biliary strictures, showing potential diagnostic performance in training and validation cohorts (all AUCs > 0.9). Bcf-eccDNAs carried cancer-related mutations, which could guide treatment. EccDNA carrying miR-106a/363 cluster or miR-374b/421 cluster were proven to regulate cancer gene expression, accelerate tumor proliferation, and inhibit tumor apoptosis.

**Conclusions:**

This study profiles a comprehensive bcf-eccDNA landscape in patients with biliary strictures and offers valuable insights into eccDNA's role in bile liquid biopsy and carcinogenesis.

## Background

Extrachromosomal circular DNA (eccDNA) is a circular DNA molecule derived and independent of linear chromosomes. More recently, eccDNAs have been found to drive tumor progression and drug resistance [1, 2]. Biliary strictures are commonly malignant in adults and arise from several types of fatal malignancies, such as pancreatic cancer, cholangiocarcinoma, and metastatic tumors. However, the characteristic landscape and oncogenic role of eccDNAs in patients with malignant biliary strictures eccDNA has not been thoroughly studied.

These tumors associated with malignant biliary strictures have a poor prognosis, and the overall survival of patients with unresectable lesions is no more than 15 months [3–6]. Due to delayed symptom onset and lack of specific diagnostic markers [7–10], most patients with malignant biliary strictures are diagnosed at an advanced and inoperable stage [11]. Therefore, there is an urgent need to unveil novel oncogenic mechanisms and biomarkers for malignant biliary strictures.

Compared with other body fluids, bile is in direct contact with tumor tissues associated with malignant biliary strictures, which can minimize interfering factors in functional and biomarker exploration to the greatest extent possible. The detection of tumor-derived cell-free DNA (cfDNA) in bile can overcome the influences of tumor heterogeneity and the technical challenges associated with tissue sampling [12, 13]. As a type of cfDNA, extrachromosomal circular DNA (eccDNA), which has a covalently closed circular structure, is more resistant to exonucleases and more stable than linear cfDNA [14]. Thus, we speculated that bile cell-free eccDNAs (bcf-eccDNAs) could be promising biomarkers for the diagnosis of malignant biliary strictures. However, the cf-eccDNA profile in human bile has not yet been revealed, and bcf-eccDNA has not been explored for potential clinical application, particularly as a cancer biomarker.

To fill this gap, we analyzed bcf-eccDNA from 40 patients with biliary strictures and demonstrated malignant-specific bcf-eccDNA profiles. To our knowledge, our work systematically characterizes the features of cf-eccDNA in human bile for the first time, reports a framework to utilize bcf-eccDNA as a potential biomarker for patients with malignant biliary strictures, and provides a valuable resource for future studies of oncogenic eccDNAs.

## Materials and methods

### Subject recruitment and sample collection

This study was approved by the Peking University Third Hospital Medical Science Research Ethics Committee (Agreement Number: M2022418). Patients who were suspected of biliary strictures and had indications of endoscopic retrograde cholangiopancreatography (ERCP) were included at Peking University Third Hospital. The patients who failed to undergo ERCP or were lost to follow-up within 1 year were excluded. All the subjects (Supplementary Table 1) provided written informed consent. The final clinical diagnoses were confirmed after a follow-up time of 1 year according to the consensus of two gastrointestinal clinicians. According to the manufacturer's instructions, the serum carcinoembryonic antigen (CEA) and carbohydrate antigen 19-9 (CA19-9) levels were analyzed via electrochemiluminescence immunoassay (Roche Diagnostics). ERCP-obtained bile specimens were centrifuged at 3000 × g for 10 min at 4 °C, and the supernatants were stored at −80 °C.

### Purification, sequencing, and identification of bcf-eccDNA

CfDNA was extracted from 0.5 mL of bile specimen using the MagMAX^™^ Cell-free DNA Isolation Kit (Thermo Scientific, A29319). EccDNA enrichment and sequencing were performed according to a previously described workflow [15]. Linear DNA was completely digested by treatment with 10 U of exonuclease III (NEB, M0206L) and 5 U of lambda exonuclease (NEB, M0262L) at 37 °C for 2 h. Circular DNAs (> 50 bp) were enriched using SPRI beads (Beckman Coulter, a63881) with a size cutoff (2 beads:1 sample). After rolling circle amplification by phi29 DNA polymerase (NEB, M0269L) and debranching by T7 endonuclease I (NEB, M0302L), the nanopore sequencing library for eccDNA was prepared using a ligation sequencing kit and sequenced on a PromethION platform according to the manufacturer’s instructions. For eccDNA identification, the raw reads were mapped to the human genome (*hg38*) using minimap2 software (version 2.18). EccDNA was generated from the mapped reads (the number of tandem repeat sequences $\ge$ 2) using eccDNA_RCA_nanopore software.

### Genomic distribution of eccDNA

EccDNA data were aligned to the *hg38* reference genome using minimap2 software (version 2.18). The coverage of eccDNA fragments at each window (1,000,000 bp) of the genome was calculated using bedtools software (version 2.30.0). After normalizing coverage, the median distribution of small eccDNA fragments across each chromosome was plotted using RIdeogram R package (version 0.2.2).

### Genomic element annotation of eccDNA, enrichment analysis of genes carried by eccDNA, and evaluation of eccDNA carrying gene (eccGene) as diagnostic biomarkers

The genomic element annotation of eccDNA was performed with HOMER software (version 4.11). EccDNAs carrying exons or introns were defined as eccGenes. Differential eccGenes between the cancer group and the noncancer group were assessed by edgeR R package (version 3.32.1) according to the criteria of at least a twofold change and *padj* < 0.05. The KEGG pathway enrichment analysis of the genes whose sequences were carried by these differential eccGenes was performed using DAVID online tool. As previously described [15], the proportion of differential eccGenes was used for cancer diagnosis, and the diagnostic value was evaluated by the area under the receiver operating characteristic curve (AUC). Using the boot.roc algorithm in the fbroc R package, the receiver operating characteristic curve was generated by bootstrapping for 2000 replicates. The optimal cutoff point was determined using Youden’s index.

### Identification and validation of single nucleotide variants (SNVs) in eccDNA

Based on the human reference genome (*hg38*), variants in eccDNA were called using Longshot software (version 0.4.1). Cancer-related annotation was performed with the COSMIC tool (version 98). The significance of the mutations in the COSMIC database was divided into five tiers. Among them, Tier 1, Tier 2, and Tier 3 mutations were related to cancer. Primer sets were designed to amplify regions containing SNV sites. The base composition of the polymerase chain reaction (PCR) products was confirmed by Sanger sequencing.

### EccEnhancer (eccDNA carrying intact super-enhancer) annotation

Based on the super-enhancer data downloaded from SEdb 2.0, eccDNA that carries an intact super-enhancer and the closest active genes regulated by the corresponding super-enhancer were annotated. KEGG pathway enrichment analyses of the target genes were performed via DAVID online tool. Using the web tool GEPIA [16], the expression levels of the target genes and the overall survival rate analysis were calculated in a cancer cohort associated with malignant biliary strictures from The Cancer Genome Atlas (TCGA), which included liver hepatocellular carcinoma (LIHC), cholangiocarcinoma (CHOL), colon adenocarcinoma (COAD) and pancreatic adenocarcinoma (PAAD) cohorts.

### EccDNA carrying intact microRNA gene (eccMIR) analysis and cancer gene annotation

EccDNAs were mapped to microRNAs (miRNAs) downloaded from Ensembl BioMart, and each eccDNA that carries an intact microRNA gene was defined as an eccMIR. The overall survival rate analysis of miRNAs was performed with the web tool Kaplan–Meier Plotter [17]. KEGG pathway enrichment analyses of miRNAs were performed via DAVID online tool. The annotation of miRNA clusters was performed using the web tool TAM 2.0 [18]. Each eccDNA carrying an intact miRNA cluster was defined as an eccMIR cluster. The annotation of cancer genes, including oncogenes and tumor suppressors, was performed with NCG 7.1 software [19].

### EccDNA synthesis using a novel method called gibson assembly large-circle accumulation (GALA)

In the GALA method (Supplementary Fig. 1), linear fragments corresponding to the targeted eccDNA were amplified from human genomic DNA using GoldenStar T6 Super PCR Mix Ver.2 (Tsingke, TSE102). Then, PCRs were performed to generate linear fragments, which had the same ends (20 bp) as the adjacent fragment (in the targeted eccDNA). These PCR products with complementary ends were mixed and incubated with 1X NEBuilder HiFi DNA Assembly Master Mix (NEB, E2621X) for 60 min at 55 °C to generate the targeted eccDNA. Using the eccDNA product as a template, two linear dsDNA molecules that complement each other in a split-reversed way were generated via PCR. These linear DNA molecules were mixed at a 1:1 molar ratio and then incubated with 80 U of Taq DNA ligase (NEB, M0208L) (95 °C for 5 min; followed by 10 cycles of 95 °C for 20 s, 4 °C for 1 min, and 45 °C for 20 min). Linear DNA was digested with 20 U of exonuclease III (NEB, M0206L) and 10 U of lambda exonuclease (NEB, M0262L) at 37 °C for 1 h. DNA gel extraction (TIANGEN, DP214) of the target fragment was used to purify eccDNA. The junctional position of the final purified eccDNA was verified using Sanger sequencing.

For eccDNA controls, a 2.5 kb random DNA sequence with 50% guanine-cytosine content was generated by the web tool “Random DNA sequence generator” (http://www.faculty.ucr.edu/~mmaduro/random.htm), and its linear fragment was synthesized by BGI. EccDNA carrying random DNA sequence (eccRandom) was synthesized using the GALA method.

### EccDNA transfection

HepG2 cells (ATCC) were cultured in DMEM supplemented with 10% FBS (HyClone, SV30208.02) in a humidified incubator containing 5% CO_2_ at 37 °C. HepG2 cells were seeded in 12-well plates and cultured for 24 h before transfection with Lipo8000 (Beyotime, C0533) and 500 ng of synthetic eccDNA according to the manufacturer’s instructions. After 48 h, the cells were harvested for subsequent analysis. Cells transfected with eccRandom were used as the control group.

### RNA-seq analysis and RT-qPCR assay of miRNAs and target mRNAs

Total RNA was isolated with TRIzol^™^ reagent (Invitrogen, 15596018). A Dynabeads^™^ mRNA Purification Kit (Invitrogen, 61006) was used to purify the mRNAs. The DNA library was prepared and sequenced on the DNBSEQ-T7 platform (MGI) with 150-bp paired-end reads. Clean reads were mapped to the reference genome (*hg38*) using STAR Aligner (version 2.7.0). StringTie (version 1.3.3b) was used to calculate gene counts. Differentially expressed genes (DEGs) were analyzed using edgeR R package (version 3.32.1) and filtered based on an FDR < 0.05 and a fold change > 2. The GO and KEGG enrichment analyses of DEGs were performed with the DAVID online tool.

For the miRNA assay, cDNA was synthesized using miRNA 1st Strand cDNA Synthesis Kit (Vazyme, MR101-01), and the qPCR assay was conducted using miRNA Universal SYBR qPCR Master Mix (Vazyme, MQ101-02). The expression level of miRNA gene was normalized to that of the *U6* gene.

For the mRNA assay, cDNA was synthesized using HiScript III 1st Strand cDNA Synthesis Kit (Vazyme, R312-02), and qPCR was performed using ChamQ Universal SYBR qPCR Master Mix (Vazyme, Q711-03). The expression level of mRNA gene was normalized to that of the *GAPDH* gene.

All primers used were listed in Supplementary Table 2. The relative expression levels of miRNAs and mRNAs were calculated with the 2^−ΔΔCt^ method.

### Cell proliferation assays

After transfection, the HepG2 cells (1 × 10^4^) were seeded into 96-well microplates. After 2 days, CCK-8 reagent (Beyotime, C0038) was added to each well, and the cells were incubated at 37 °C for 1–2 h. The absorbance at 450 nm was recorded by a configurable multimode microplate reader (Synergy H1, BioTek).

### Cell apoptosis detection

After transfection, the HepG2 cells (5 × 10^5^) were stained with Annexin V-FITC and PI solution using an apoptosis kit (Vazyme, A211-02). Apoptotic cells were detected with a FACSCanto system (BD Biosciences) and analyzed using FlowJo software.

### Statistical analysis

Samples conforming to a normal distribution and those not conforming to a normal distribution were compared by two-tailed unpaired *t* test and the Wilcoxon signed-rank test, respectively, using SPSS software (version 25.0). P < 0.05 indicated statistical significance.

## Results

### Larger size, six characteristic peaks, and origin preference of bcf-eccDNA in the malignant biliary strictures

**Fig. 1:**
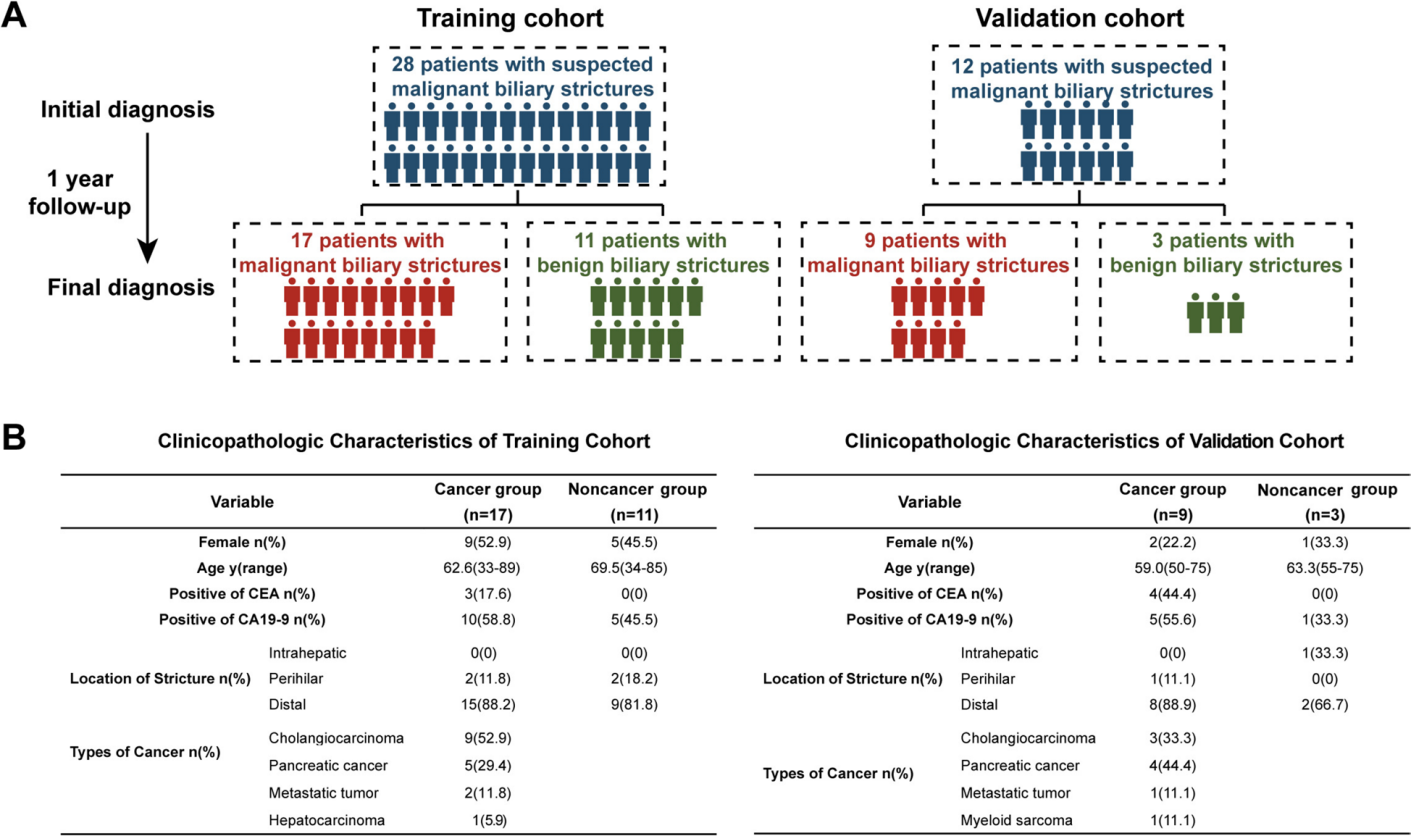
Diagnoses and characteristics of the training cohort and the validation cohort. **A** Flow chart indicating the initial and final clinical diagnoses of the patients. **B** Clinicopathologic characteristics of the two cohorts at the time of initial diagnosis and their final cancer-related diagnosis. *CEA* carcinoembryonic antigen, *CA19-9* carbohydrate antigen 19–9

We recruited a cohort of 28 patients who underwent ERCP for suspected malignant biliary strictures; these patients were ultimately classified as having strictures of benign (n = 11) or malignant origin (n = 17) after 1 year of follow-up (Fig. 1). Another independent cohort for validation was subsequently recruited and included 9 patients with malignant biliary strictures and 3 patients with benign biliary strictures (Fig. 1).

**Fig. 2:**
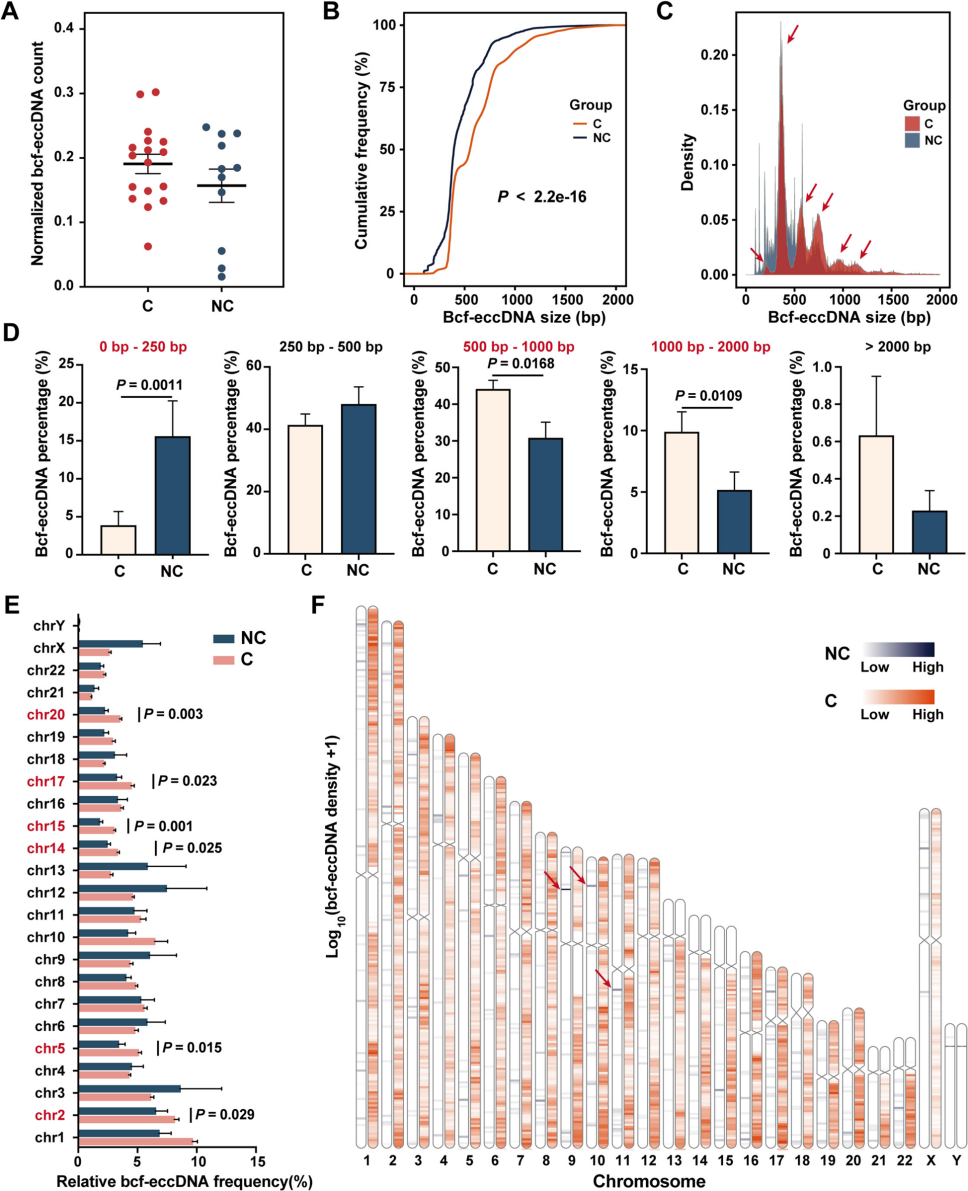
Different hallmarks of the bcf-eccDNA profile in the cancer group (**C**) and the noncancer group (NC). **A** The bcf-eccDNA count per mapped read. **B** A cumulative frequency plot of bcf-eccDNA (shorter than 2000 bp). **C** The size distribution of bcf-eccDNA (shorter than 2000 bp). **D** Comparison of bcf-eccDNA within different length intervals between the cancer group and the noncancer group. **E** The chromosomal distribution of bcf-eccDNA. **F** The genomic distribution of bcf-eccDNA

As expected, cf-eccDNA was commonly present in human bile. A median of 79,602 (95% confidence interval [CI], 42,280–97,055) bcf-eccDNAs were identified in the cancer group, while 4,956 (95% CI, 481–70,753) were identified in the noncancer group. After normalization by sequencing depth, the numbers of eccDNAs identified in noncancer and cancer bile samples were comparable (Fig. 2A). To avoid potential influences of sequencing depth between different samples, mapped reads were downsampled to the same for subsequent analysis.

Nearly all (~ 99%) of the bcf-eccDNAs were shorter than 2000 bp in both groups. Interestingly, bcf-eccDNA in the cancer group was significantly longer than that in the noncancer group (Fig. 2B), and the overall size distributions of the two groups were different (Fig. 2C). Importantly, compared to those in the noncancer group, the size of bcf-eccDNAs (0–2000 bp) in the cancer group was enriched in six peaks, at ~ 200 bp, ~ 380 bp, ~ 560 bp, ~ 740 bp, ~ 950 bp, and ~ 1150 bp. In an independent validation cohort, these six characteristic peaks were observed in all patients with malignant biliary strictures and none of the patients with benign biliary strictures (Supplementary Fig. 2). Compared with those in the noncancer group, more bcf-eccDNA within 500–1000 bp or 1000–2000 bp and fewer bcf-eccDNA within 0–250 bp were detected in the cancer group (Fig. 2D). The eccDNA in the cancer group was larger, possibly because cancer cells are prone to produce relatively larger eccDNAs and release them into bile [20].

Compared with those in the noncancer group, bcf-eccDNA in the cancer group more frequently originated from chromosomes (Chr) 2, 5, 14, 15, 17, and 20 (all *P* $<$ 0.05) (Fig. 2E). Because of the positive correlation between transcription activity and eccDNA generation [14], genes on these chromosomes may host higher transcription activity in patients with malignant biliary strictures. In addition, bcf-eccDNA in the noncancer group showed locus preferences (Fig. 2F). But bcf-eccDNA in the cancer group was randomly derived from the whole genome, reflecting increased genomic instability in cancer.

### Differential bile cell-free eccGenes (bcf-eccGenes) with high diagnostic accuracy for malignant biliary strictures

**Fig. 3:**
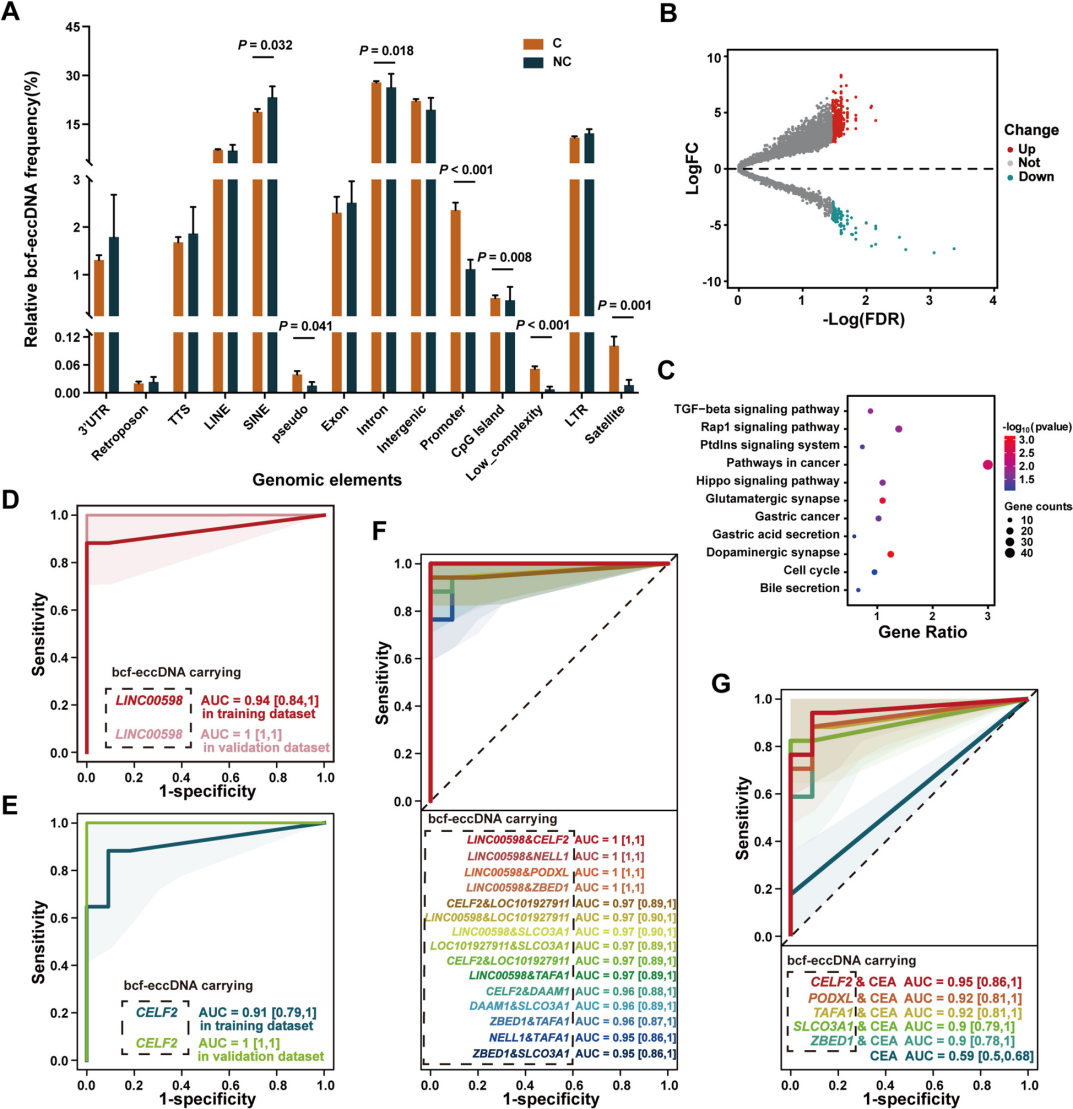
Genomic annotation of bcf-eccDNA in the cancer group (**C**) and the noncancer group (NC). **A** Genomic elements carried by bcf-eccDNA. **B** Volcano map of the differential bcf-eccGenes between the cancer group and the noncancer group. **C** KEGG pathway enrichment of differential bcf-eccGenes. **D** The diagnostic performance of bcf-eccDNA carrying *LINC00598* is assessed in the training cohort and subsequently validated in the validation cohort. **E** The diagnostic performance of bcf-eccDNA carrying *CELF2* is assessed in the training cohort and subsequently validated in the validation cohort. **F** Diagnostic performance of the combination of two bcf-eccGenes (all AUCs ≥ 0.95). **G** The diagnostic performance of the combination of bcf-eccGenes and serum CEA levels. *AUC* area under the receiver operating characteristic curve, *CEA* carcinoembryonic antigen

To study the functions of eccDNA in the cancer group, we investigated the genomic elements carried by bcf-eccDNA. Notably, we detected a different profile of genomic elements in the cancer group than in the noncancer group, especially for CpG islands and promoters, which have been reported to play crucial roles in regulating gene transcription (Fig. 3A) [21]. Moreover, we found 1425 significantly upregulated and 165 significantly downregulated bcf-eccGenes in the cancer group (Fig. 3B). Genes carried by these differential bcf-eccGenes were associated with several important cancer-related pathways, such as the hippo signaling pathway and TGF − beta signaling pathway (Fig. 3C) [22–24]. Because eccGenes can be transcribed into intact or truncated genes [25], these bcf-eccGenes may activate cancer-related pathways to play a role in carcinogenesis.

The bcf-eccDNA features in the cancer group had the potential to serve as meaningful biomarkers. Thus, we investigated the diagnostic value of all differential bcf-eccGenes between the cancer group and the noncancer group. Strikingly, bcf-eccDNAs carrying *LINC00598* or *CELF2* demonstrated high diagnostic performance for identifying malignant biliary strictures, with an AUC of 0.94 (95% CI, 0.84–1) and an AUC of 0.91 (95% CI, 0.79–1), respectively (Fig. 3D, E). Furthermore, their diagnostic performance was strongly confirmed in the validation cohort (Fig. 3D, E). Moreover, the cutoff points for bcf-eccDNA carrying *LINC00598* (0.0063%) and bcf-eccDNA carrying *CELF2* (0.0029%) also exhibited high diagnostic accuracy (100% and 75%, respectively) in the validation cohort. In addition, the combination of two differential bcf-eccGenes increased the AUC to $\ge$ 0.95, especially for the combination of bcf-eccGenes carrying *LINC00598* with bcf-eccGenes carrying *CELF2/NELL1/PODXL/ZBED1* (Fig. 3F). Moreover, the diagnostic performance of CEA (AUC = 0.59) or CA19-9 (AUC = 0.6) in serum was increased (AUC $\ge$ 0.9), when combined with bcf-eccGenes (Fig. 3G and Supplementary Fig. 3).

### Bcf-eccDNA harbors SNV information

Variants in bile linear cfDNA provide a source of diagnostic biomarkers for malignant biliary strictures [13]. However, linear cfDNA is often fragmented and structurally unstable, leading to a high degradation rate [26]. We found that there were no cancer-related SNVs in the bcf-eccDNA from the noncancer group. More importantly, we identified three genes with Tier 3 SNVs in patients with malignant biliary strictures, namely, *RUNDC1* (g.T42981054C), *SLC16A2* (g.T74421734C), and *ANKLE1* (g.G17282085A) (Supplementary Fig. 4A). Their mutation frequencies in cancer samples included in the COSMIC project were 0.046, 0.024, and 0.043, respectively. In addition, we also identified several SNVs of unknown clinical significance in bcf-eccDNA carrying cancer genes in both groups (Supplementary Fig. 4B). This observation is because spontaneously occurring mutations accumulate in somatic cells throughout a person's lifetime, but most mutations do not have a noticeable effect [27]. To determine the reliability of the detected SNVs, we randomly selected one Tier 3 SNV and four SNVs in cancer-related genes for outward PCR and Sanger sequencing. As expected, all SNVs in bcf-eccDNA were verified (Supplementary Fig. 4C).

### Cancer-specific eccEnhancers may contribute to cancer development and poor outcomes

**Fig. 4:**
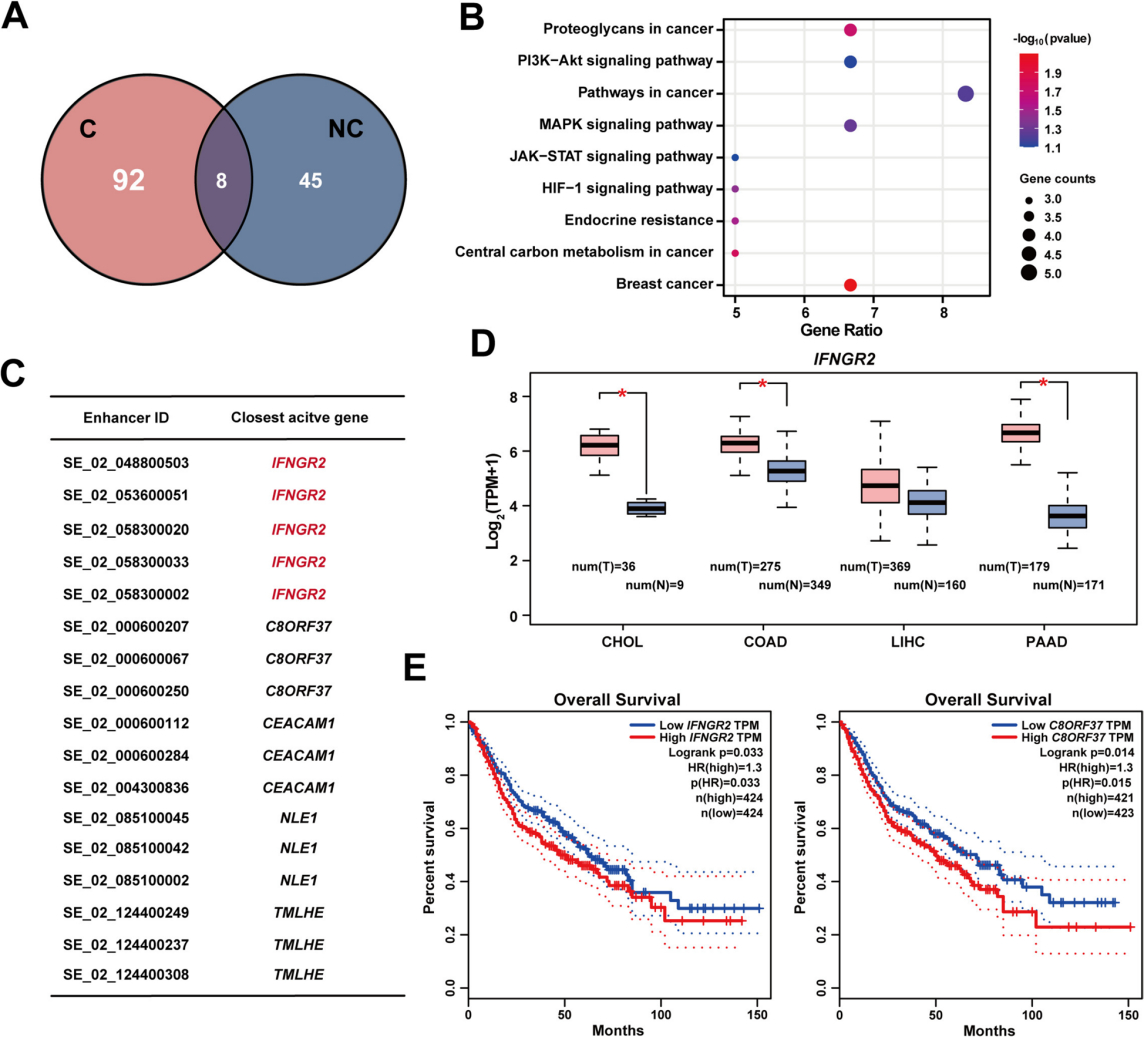
Cancer-specific eccEnhancers contribute to cancer development and poor outcomes. **A** The different eccEnhancers in the cancer group (**C**) and the noncancer group (NC). **B** KEGG pathway enrichment of the closest active genes regulated by super-enhancers carried by cancer-specific eccEnhancers. **C** The target genes are regulated by multiple (≥ 3) super-enhancers carried by cancer-specific eccEnhancers. **D** Expression levels of *IFNGR2* in the CHOL, COAD, LIHC, and PAAD cohorts from the TCGA database. *P < 0.05. **E** Overall survival analysis according to *IFNGR2* and *C8ORF37* expression levels in cancer cohorts associated with malignant biliary strictures from the TCGA database, including CHOL, COAD, LIHC, and PAAD cohorts. *PAAD* pancreatic adenocarcinoma, *CHOL* cholangiocarcinoma, *COAD* colon adenocarcinoma, *LIHC* liver hepatocellular carcinoma

EccEnhancers can interact with chromosomes as mobile super-enhancers to activate chromosomal transcription [28]. The profiles of the eccEnhancers differed between the cancer group and the noncancer group, with only 5.5% of the shared eccEnhancers, and 92 eccEnhancers specifically detected in the cancer group (Fig. 4A). This diferences indicated that cancer cells may have unique transcriptional regulatory mechanisms. To further explore the functions of these cancer-specific eccEnhancers, we annotated the closest active genes of super-enhancers carried by cancer-specific eccEnhancers and found that these target genes were enriched in some cancer-related pathways, such as the PI3K-Akt signaling pathway, MAPK signaling pathway and pathways in cancer (Fig. 4B) [29, 30]. These target genes included some oncogenes, such as *MYC* [31] and *EGFR* [32] (Supplementary Table 3). These results indicated that cancer-specific eccEnhancers may play a role in tumorigenesis and cancer evolution.

Among these closest active genes of super-enhancers carried by cancer-specific eccEnhancers, we explored the target genes regulated by multiple ($\ge$ 3) super-enhancers (Fig. 4C). These genes, especially *IFNGR2*, were significantly overexpressed in some TCGA cohorts associated with malignant biliary strictures (Fig. 4D and Supplementary Fig. 5), which was in line with the finding that eccEnhancers can activate genome-wide transcriptional amplification [28, 33]. Moreover, the expression levels of *IFNGR2* and *C8ORF37* were both significantly negatively correlated with the prognosis of patients with cancer associated with malignant biliary strictures, suggesting that eccEnhancers that activate *IFNGR2* or *C8ORF37* may contribute to poor outcomes (Fig. 4E).

### Cancer-specific eccMIRs regulate cancer gene expression and accelerate tumor proliferation

**Fig. 5:**
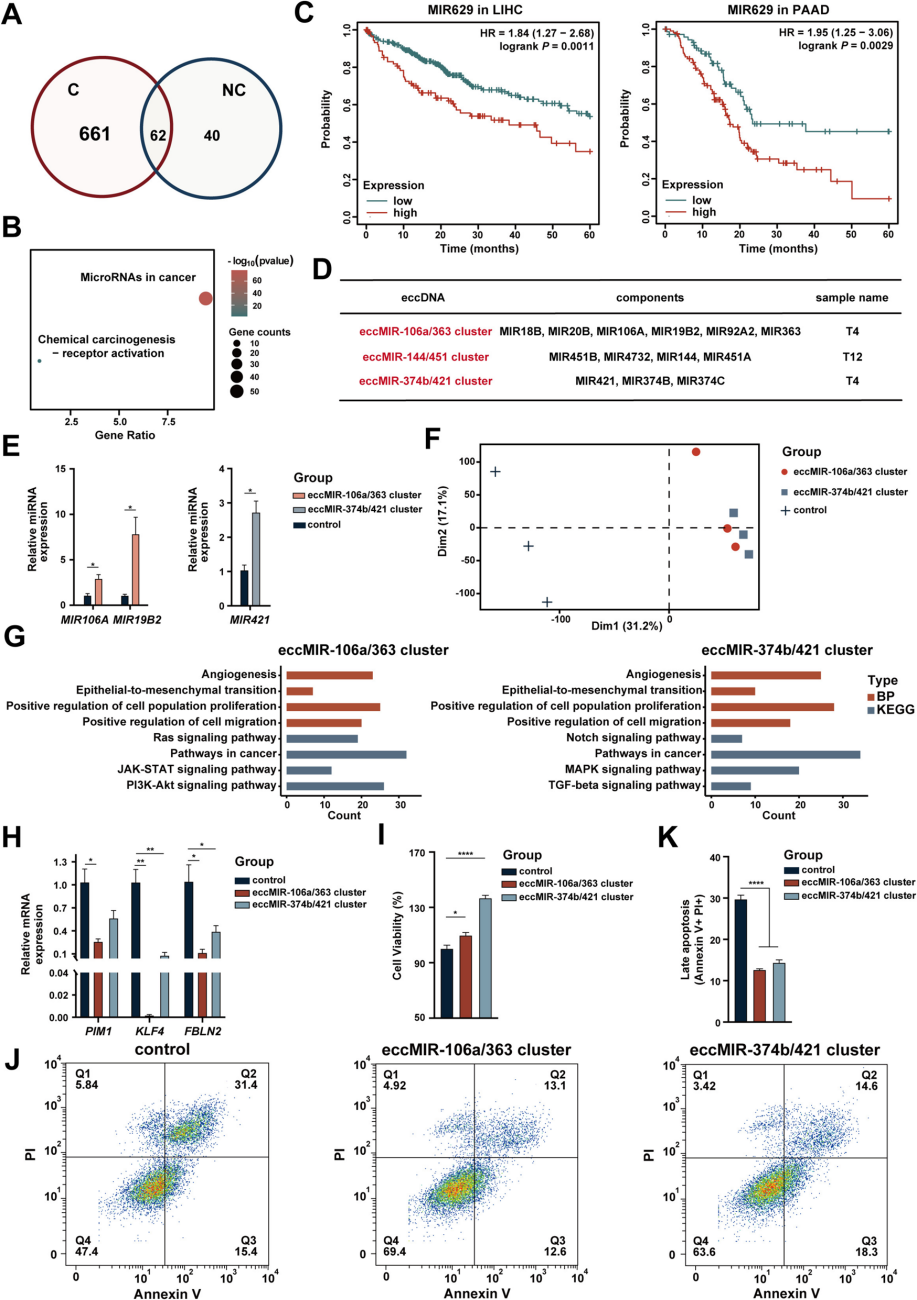
Cancer-specific eccMIR clusters produced functional miRNAs and promoted tumor proliferation. **A** The different eccMIRs in the cancer group (**C**) and the noncancer group (NC). **B** KEGG pathway enrichment of miRNAs carried by cancer-specific eccMIRs. **C** Overall survival analysis according to the MIR629 expression level in the LIHC and PAAD cohorts from the TCGA database. **D** Two eccMIR clusters are detected specifically in the cancer group. **E** Two eccMIR clusters elevate the endogenous miRNA levels. **F** The PCA plot shows the difference in expression profiles after the treatment of the two eccMIR clusters. **G** KEGG pathway and biological process (BP) enrichment of the DEGs after treatment of the two eccMIR clusters. **H** The expression levels of endogenous cancer genes are reduced by the two eccMIR clusters. **I** Cell viability is increased by the two eccMIR clusters. **J**, **K** Flow cytometry-based detection of apoptosis and the percentage of cells undergoing late apoptosis in each group. *P < 0.05, **P < 0.01, ***P < 0.001 and ****P < 0.0001. *LIHC* liver hepatocellular carcinoma, *PAAD* pancreatic adenocarcinoma

There were different eccMIR profiles between the cancer group and the noncancer group and a total of 661 cancer-specific eccMIRs were found (Fig. 5A). EccMIRs can produce functional miRNA molecules to impact cancer growth and progression [20, 34]. Therefore, we investigated the miRNAs carried by these cancer-specific eccMIRs and found that these miRNAs were enriched in several cancer-related pathways (Fig. 5B), indicating that these cancer-specific eccMIRs may play crucial roles in carcinogenesis. EccDNA carrying MIR629 (eccMIR629) was detected in five bile samples from the cancer group, and MIR629 was significantly associated with a lower overall survival rate in the LIHC and PAAD cohorts (Fig. 5C). These findings suggest that eccMIR629 may serve as a poor prognostic biomarker.

Strikingly, three eccDNAs carrying the intact miRNA clusters (eccMIR clusters) were specifically found in cancer patients, including eccDNA carrying miR-106a/363 cluster (eccMIR-106a/363 cluster), eccDNA carrying miR-144/451 cluster (eccMIR-144/451 cluster), and eccDNA carrying miR-374b/421 cluster (eccMIR-374b/421 cluster) (Fig. 5D and Supplementary Fig. 6). MiRNA clusters contain multiple coexpressed miRNAs that can play a crucial role synergistically and individually and have more powerful regulatory capabilities than a single miRNA [35–37]. However, the functions of eccMIRs, especially in carcinogenesis, are unclear.

To further verify the function of eccMIRs, we developed a novel method named GALA, which has the capacity to generate large eccDNAs derived from any genomic region, particularly multiple genomic regions (Supplementary Fig. 1). As shown in Supplementary Fig. 1, the eccMIR-106a/363 cluster, eccMIR-144/451 cluster, and eccMIR-374b/421 cluster were successfully synthesized by the GALA method. Given that the eccMIR-106a/363 cluster and eccMIR-374b/421 cluster were identified in one patient with hepatocarcinoma (Fig. 5D and Supplementary Table 1), we transfected each of them into HepG2 cells to investigate their impact on hepatocarcinoma. We found that these eccMIR clusters could significantly upregulate the expression levels of their corresponding miRNAs (Fig. 5E).

Furthermore, we systematically characterized their expression profiles via an RNA-seq assay (Fig. 5F). After treatment with the eccMIR-106a/363 cluster or eccMIR-374b/421 cluster, DEGs were enriched in several cancer-associated signaling pathways, including the PI3K-Akt, MAPK, and Notch pathways (Fig. 5G). In addition, several biological features such as proliferation and epithelial-to-mesenchymal transition, were also regulated by these two eccMIR clusters. More importantly, these two eccMIR clusters significantly regulated the expression levels of several cancer genes, indicating that eccMIR plays a crucial role in carcinogenesis and presents the ability to regulate cancer progression (Supplementary Fig. 7). Among all overlapping cancer genes regulated by these two eccMIR clusters, the downregulated expression of *PIM1*, *KLF4*, and *FBLN2* was confirmed via RT-qPCR (Fig. 5H). The eccMIR-106a/363 cluster and eccMIR-374b/421 cluster significantly increased proliferation and reduced late apoptosis in HepG2 cells (Fig. 5I, J).

## Discussion

Over the past few years, considerable interest has been focused on the exploration of eccDNA features in tissue, plasma, and urine [14, 15, 38]. However, its presence and value in bile and its role in malignant biliary strictures are still largely unclear. Our current study demonstrated that eccDNA was ubiquitous in human bile, and we systematically profiled bile cell-free eccDNA (bcf-eccDNA) characteristics in patients with malignant biliary strictures. The feature of bcf-eccDNA provided valuable information for disease studies on biomarkers and pathogenesis.

We identified tens of thousands of bcf-eccDNAs, and these molecules in the cancer group were widely and evenly distributed across the human genome. The size of bcf-eccDNA in the cancer group was larger than that in the noncancer group, which was in line with the large eccDNA commonly observed in cancer cells [39]. Larger eccDNA can carry longer genomic segments, providing a larger genetic reservoir for the rapid evolution of cancer. Besides, we observed six peaks of bcf-eccDNA distribution in the cancer group for the first time. These six peaks are consistent with those of a cancer cell line [40]. Two larger peaks, ~ 950 bp and ~ 1150 bp, were not observed in other body fluids from cancer patients, such as plasma [15]. We speculate that this is because bile directly contacts tumor tissues, which contributes to reflecting the instant eccDNA profile of cancer cells. In addition, compared with that in the noncancer group, the generation of bcf-eccDNA in the cancer group favored CpG islands and promoters. CpG islands in cancer cells exhibit widespread hypermethylation, which can inhibit the cleavage rates of DNA by nucleases [41, 42]. This may be the reason why there were more eccDNA carrying CpG islands in the cancer group than in the noncancer group. Compared with the chromosomal promoter, the promoter on eccDNA exhibits hypomethylation [43], suggesting that the favored eccDNA carrying promoters may enable more active transcription in patients with malignant biliary strictures. Considering that the formation of eccDNA is associated with genomic instability [44], we speculate that CpG islands and promoters are highly unstable in cancer patients.

As circular DNA molecules, cf-eccDNAs are structurally more stable than cell-free linear DNA and RNA and are therefore more promising biomarkers. We observed that bcf-eccDNA carrying *LINC00598* or *CELF2* had high diagnostic accuracy in cancer screening. *LINC00598* plays an important role in cell cycle regulation and proliferation [45]. *CELF2* serves as a tumor suppressor gene and prognostic biomarker [46, 47]. We observed that the serum CEA and CA19-9 levels had limited disease specificity and were ineffective in cancer screening or diagnosis, which was consistent with the findings of previous studies [48]. However, when CEA/CA19-9 was combined with eccGenes, the diagnostic performance for malignant biliary strictures was remarkably improved. In the future, we will expand the sample size to validate the diagnostic robustness of the eccDNA-based biomarkers.

Interestingly, similar to other cell-free molecules [49], bcf-eccDNA carries mutation information, indicating that cf-eccDNA has potential as a reliable biomarker in clinical scenarios, such as for characterizing resistance mechanisms to targeted therapies and informing sequential therapy. Moreover, bile-based eccDNA analysis can provide cancer-related SNV information, which cannot be detected by plasma-based eccDNA analysis [50]. This is possibly because of the close physical proximity of bile to premalignant or malignant cells in the bile duct system and because mutations in plasma cfDNA are mainly from white blood cells rather than from cancer cells [51]. Our future studies will focus on exploring the relationship between mutations in bcf-eccDNA and somatic mutations in cancer tissues to clarify the clinical significance of mutations in bcf-eccDNA.

Moreover, extensive exploration of eccDNA functions has been limited due to the lack of efficient and robust methods for synthesizing eccDNA. The ligase-assisted minicircle accumulation (LAMA) approach has limited usage in the synthesis of large eccDNA (> 100 bp) [52]. QuickLAMA requires seven steps and can synthesize only eccDNA containing a single genomic region [53]. Our novel method, Gibson Assembly large-circle accumulation (GALA), provides a simple and efficient workflow for synthesizing large eccDNAs containing any genomic region within five steps. Gibson assembly can efficiently assemble multiple fragments in the correct order. Therefore, the GALA approach can easily synthesize eccDNA derived from multiple genomic regions without the need for additional steps. Thus, the GALA approach provides a powerful workflow for the scientific and clinical study of eccDNA.

The eccDNAs carrying the intact miRNA clusters (eccMIR clusters) which contain multiple coexpressed miRNAs have more powerful regulatory capabilities than a single eccMIR. The two eccMIR clusters, eccMIR-106a/363 cluster and eccMIR-374b/421 cluster were specifically observed in the cancer group. We synthesized these eccMIR clusters using the GALA approach and systematically explored their functions in oncogenesis. These eccMIR clusters can produce corresponding miRNAs, regulate cancer gene expression, and affect cancer-related features and signaling pathways. Among these cancer genes, *KLF4* suppresses the proliferation of cholangiocarcinoma [54], while *FBLN2* is an independent protective factor for hepatocarcinoma [55]. In addition, we further confirmed that they can significantly accelerate tumor proliferation and inhibit tumor apoptosis; thus, blocking the production of these eccMIR clusters may constitute a novel therapeutic strategy. These findings provide insight into tumor pathogenesis and cancer therapy, as well as into eccDNA and miRNA biology.

This study has several limitations. Although it’s easy to collect bile samples during ERCP, ERCP operations require a skillful endoscopist. Complex components in bile have the potential to increase the instability of DNA. The burden of bioinformatics analysis of eccDNA may also limit clinical applications. Besides, the sample size was relatively small. In the future, we will conduct prospective, large-sample, multicenter trials to determine the diagnostic capability of eccDNA-based biomarkers in patients with suspected biliary strictures and further verify the role of eccDNA in biliary neoplasms.

## Conclusions

In summary, we systematically debuted the features of cf-eccDNA in human bile, reported a framework to leverage bcf-eccDNA as a potential biomarker for patients with malignant biliary strictures, and provided comprehensive baseline information for future studies of cancer-specific biomarkers. Furthermore, a simple and efficient workflow was developed to synthesize large eccDNA particularly containing multiple regions, which greatly advances the functional exploration of eccDNA.

## Data Availability

Sequencing data can be downloaded from https://ngdc.cncb.ac.cn/omix (accession No. OMIX005861).

## Abbreviations

eccDNA: Extrachromosomal circular DNA
cfDNA: Cell-free DNA
cf-eccDNA: Cell-free eccDNA
bcf-eccDNA: Bile cell-free eccDNA
eccGene: EccDNA carrying gene
bcf-eccGene: Bile cell-free eccGene
eccEnhancer: EccDNA carrying intact super-enhancer
miRNA: MicroRNA
eccMIR: EccDNA carrying intact miRNA gene
eccMIR cluster: EccDNA carrying intact miRNA cluster
eccRandom: EccDNA carrying random DNA sequence
TCGA: The Cancer Genome Atlas
PAAD: Pancreatic adenocarcinoma
LIHC: Liver hepatocellular carcinoma
CHOL: Cholangiocarcinoma
COAD: Colon adenocarcinoma
AUC: Area under the receiver operating characteristic curve
CEA: Carcinoembryonic antigen
CA19-9: Carbohydrate antigen 19-9
DEG: Differentially expressed gene
